# Loss of muscle mass in the immediate post-operative period is associated with inadequate dietary protein and energy intake

**DOI:** 10.1038/s41430-023-01264-0

**Published:** 2023-01-26

**Authors:** E. J. Hardy, C. S. Deane, J. N. Lund, B. E. Phillips

**Affiliations:** 1 Department of General Surgery, Royal Derby Hospital, Derby, UK; 2 Centre Of Metabolism, Ageing and Physiology, University of Nottingham, Royal Derby Hospital Centre, Derby, UK; 3 Nottingham NIHR Biomedical Research Centre and MRC/Versus Arthritis Centre for Musculoskeletal Ageing Research, Nottingham, UK; 4 Department of Sport and Health Sciences, College of Life and Environmental Sciences and Living Systems Institute, University of Exeter, Exeter, UK; 5 Human Development & Health, Faculty of Medicine, University of Southampton, Southampton General Hospital, Southampton, UK

**Keywords:** Nutrition, Translational research

## Abstract

Despite the implementation of ‘Enhanced Recovery After Surgery’ (ERAS) protocols, major abdominal surgery is still associated with significant and detrimental losses of muscle mass and function in the post-operative period. Although ERAS protocols advocate both early mobility and dietary intake, dietary composition in the immediate post-operative period is poorly characterised, despite muscle losses being greatest in this period. Herein, we show in 15 patients (66 ± 6 y, 12:3 M:F) who lost ~10% *m. vastus lateralis* muscle mass in the 5 days after open colorectal resective surgery, mean energy intake was only ~25% of the minimum ESPEN recommendation of 25 kcal/kg/d and daily dietary protein intake was only ~12% of the ESPEN recommended guidelines of 1.5 g/kg/d. Given the known importance of nutrition for muscle mass maintenance, innovative dietary interventions are needed in the immediate post-operative period, accounting for specific patient dietary preference to maximise compliance (e.g., soft-textured foods).

## Introduction

Substantial losses of skeletal muscle mass and function occur after major gastrointestinal (GI) surgery - likely due to the physiological insult of surgery, physical inactivity, and inadequate protein nutrition in the post-operative period [1]; with the greatest losses occurring in the first post-operative week [2]. Surgery-related muscle loss is associated with declines in muscle function important for independence, a slower return to normal activities, and reduced quality of life [3]. In addition, as the majority of major abdominal surgery is performed in patients over 60 years of age [4], these patients face this surgery and the associated muscle mass losses, on a background of sarcopenia [5]. Furthermore, in older age, the cumulative effect of repeated short bouts of muscle disuse (such as those associated with surgery), may be a key factor in the development of frailty [3], which incurs significant burden to individuals, families, and society.

Mass muscle maintenance is regulated by the dynamic processes of muscle protein synthesis (MPS) and breakdown (MPB), which in healthy muscle is achieved via postprandial increases in MPS interspersed with postabsorptive periods of negative net protein balance (i.e., MPB > MPS), with contractile activity known to enhance MPS responses to nutrition [6]. In addition to promoting early mobility, ‘Enhanced Recovery After Surgery’ (ERAS) protocols aim to accelerate post-operative recovery by advocating early dietary intake. However, they do not provide specific guidance on nutritional composition [7], leading to wide variability in practice. Therefore, this study aimed to characterise the true immediate post-operative dietary energy and protein intake of abdominal surgery patients in the context of the most recent and relevant European Society for Clinical Nutrition and Metabolism (ESPEN) guidelines [8], with a view to inform future nutritional approaches to maximise muscle mass maintenance in this crucial period of recovery.

## Methods

Fifteen patients (demographics are provided in Table 1) undergoing open major colorectal resection and following an ERAS protocol at a single hospital site in the United Kingdom were recruited to this study (NHS Ethics Committee Approval: 20/EM/069, IRAS ID: 274048) which was registered on clinicaltrials.gov (NCT04199936). Dietary intake on post-operative days (POD) 1 to 4 was recorded using a questionnaire completed by the patient alone, or with a researcher as required, and cross-referenced against hospital documentation. Details of food options were provided by the hospitals catering supplier (ISS, Søborg, Denmark), with nutrient composition calculated using specialist software (Nutritics Ltd, Dublin, Ireland). Energy intake values were compared to 25–30 kcal/kg/d and protein to 1.5 g/kg/d, based on the ESPEN guidelines regarding clinical nutrition in surgery [8]. Data is presented as mean ± SD with between-day differences analysed using one-way ANOVA and Tukey post-hoc analysis (GraphPad Software, San Diego, CA).

**Table 1:** Patient demographics.

Demographics	Patients (*n* = 15)
Sex (male:female)	4:1
Age (years)	66 ± 6
Body mass index (kg/m^2^)	28 ± 4
Surgical procedure	13 rectal cancer resections 1 hepatic flexure cancer resection 1 stricturing diverticular disease resection

Data are presented as mean ± SD.

## Results

**Fig. 1:**
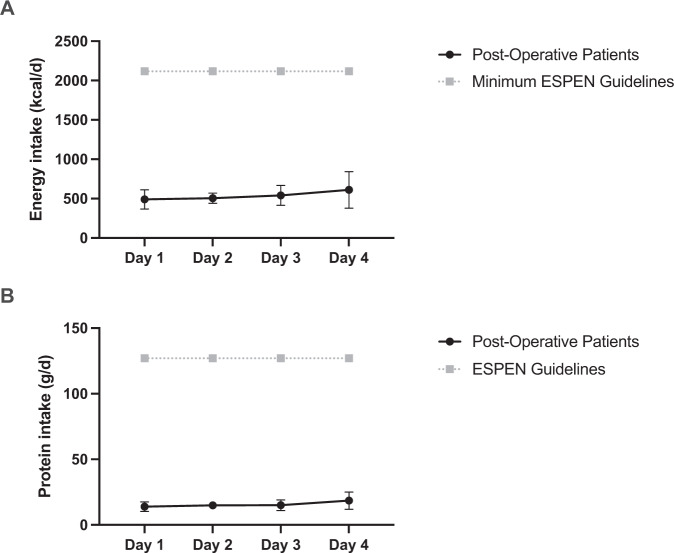
Dietary intake in post-operative patients. Mean daily energy intake (**A**) and mean daily protein intake (**B**) in post-operative patients’ 1–4 days after surgery (black data points). For daily energy intake, representative ESPEN data is calculated using the average weight of the patients recruited to this study (84.7 kg) and the minimum recommended energy intake (25 kcal/kg/d) [8]. For daily protein intake, representative ESPEN data is calculated using the average weight of the patients recruited to this study (84.7 kg) and the recommended protein intake (1.5 g/kg/d) [8]. Data are presented as mean ± SEM.

In this group of patients who lost 9.16 ± 2.0% quadriceps muscle mass (measured as *Vastus Lateralis* cross sectional area via ultrasound [9]) in the 5 days after surgery [9], mean daily energy intake was 536.6 ± 527.8 kcal across the 4 post-operative days, and 490.0 ± 470.7, 505.0 ± 251.8, 541.0 ± 488.1, 610.5 ± 900.5 kcal on POD 1 to 4 respectively (Fig. 1A). Despite a numerical increase day-on-day, there was no significant difference in energy intake between the days. Mean daily protein intake was 15.6 ± 16.4 g across the 4 post-operative days, and 13.9 ± 14.1, 14.9 ± 10.2, 15.0 ± 15.6, 18.5 ± 25.6 g on POD 1 to 4 respectively (Fig. 1B). No significant difference in protein intake was found between the days. This equated to mean daily energy intake over POD1 to 4 being just 25.3 ± 24.9% of the ESPEN recommendation, reducing to 12.8 ± 2.6% in patients with ileus. Mean daily protein intake was only 12.3 ± 12.9% of the ESPEN recommendations, and only 5.7 ± 0.9% for those with ileus. Overall, only 1 patient consumed the recommended energy intake (on POD4). No patients managed to achieve over 50% of the recommended protein intake on POD1 to 3, with just 1 patient achieving this (78%) on POD4.

Collectively for all patients, the percentage of energy consumed as protein was 11.65 ± 2.0 on POD1, 12.26 ± 4.9 on POD2, 11.12 ± 4.9 on POD3, and 11.9 ± 3.9% on POD4, indicative of a low protein diet across this period. Interestingly, compared to the day-on-day increase in energy intake across the post-operative period, the percentage of energy consumed as protein did not increase.

Patients expressed a clear preference for soft-textured foods (e.g., soup, jelly, ice cream), with these making up 73% of meal choices on POD1, 84% on POD2, 55% on POD3, and 48% on POD4. Overall, 66% of meal choices across POD1 to 4 were soft-textured.

## Discussion

In addition to encouraging early mobility, ERAS guidelines encourage early dietary intake in patients after major abdominal surgery [7]. However, this is primarily to support accelerated gastrointestinal recovery, decreased hospital length of stay, and decreased rate of complications and mortality, rather than the preservation of muscle mass and function. While early dietary intake is encouraged, we clearly demonstrate that post-operative patients are consuming well below the energy and protein requirements recommended by ESPEN [8], which may contribute to observed detrimental losses in muscle mass in these patients. Furthermore, what this post-operative diet should consist of is not specified. The importance of adequate protein intake for muscle mass maintenance is well established, however when given free choice patients appear to choose low protein, soft-texture foods, limiting anabolic potential which is likely already blunted in the face of age-associated anabolic resistance [6], post-operative inflammation and inactivity [1]. Although patients’ caloric and protein intake can be enhanced through provision of liquid supplements, these supplements are often not well-liked or tolerated [10]. As such, the development of protein-enriched forms of the whole foods popular with post-operative patients (e.g., ice-cream, jelly, soup, yogurt) may help to achieve the recommended dietary intake goals, as may more palatable liquid nutritional supplements. Both of these options should be explored to maximise protein intake and enhance skeletal muscle anabolism in these patients.

## Data Availability

The datasets generated during and/or analysed during the current study are available from the corresponding author on reasonable request.
